# Progress in Surgery of the Liver and Gall Bladder

**Published:** 1902-07-19

**Authors:** 


### PROGRESS IN SURGERY OF THE LIVER AND GALL BLADDER.

**Wounds of the Liver.**—Mercadé[1] advocates immediate interference in injury to the liver. He rejects attempts to stop bleeding from a wound in the liver by hot air, stitching the omentum into or over the wound and the thermocautery. Ligature and forcipressure are impracticable in most cases. The tampon is of proved value, but further steps are usually needed to save the patient. In superficial and almost inaccessible wounds it is useful. The suture is the best means of arresting the hæmorrhage, and provided that the edges of the wound are well approximated, the variety of suture is of secondary importance. Provided that the wound is incised and recent, the suture will stop hæmorrhage and allow of repair of the wound even in very bad cases. But in contused wounds the tampon is required. The edges cannot be made to meet; they are brcken down and friable; the suture is needed to play a secondary part, as it serves to approximate the severed liver tissue and thus aids cicatrisation which is best hastened and limited. Drainage of the wound and injection of artificial serum are needed after the application of the sutures and tampon.

**Hepatic Abscess.**—Cantlie[2] records four cases of liver abscess treated by tapping by trocar and cannula and syphon drainage (Manson's method), of which three recovered and one died. The size of the drainage tube necessary to permit of such drainage has been much exaggerated, and he believes that, with syphon drainage, the size of the tube used after tapping by the trocar and cannula will diminish as time goes on and more experience is gained. The suction obtainable by syphonage is considerable, and often errs on the side of being too severe; for along a moderately small-sized tube even thick liver pus will readily pass. The arguments in favour of the trocar and cannula are in connection with deep-seated liver abscesses and not with abscesses which actually bulge either towards the abdominal wall or the ribs, so that the pus is close below the surface. The author contends: 1. That pus should be sought for early, the moment in fact that there is a suspicion of liver abscess. 2. That it is perfectly safe, with certain precautions, to search for deep-seated pus by the hollow needle of a syringe or aspirator. 3. That it is unnecessary to expose the surface of the liver before operating. 4. Should an abscess be seen after laparotomy to bulge on the surface of the liver, it is farcical to suggest stitching it to the edges of the wound before opening, as pus is likely to escape from the pricks of the needle. 5. It is well to dissociate the operation for hydatids and for deep-seated abscesses of the liver once for all. The surgical steps in the former are not applicable in the latter. 6. The operation of transpleural hepatotomy is regarded as over-heroic. Maitland[3] and others, however, regard these views as largely erroneous. Maitland thinks that the use of the exploring needle for the purpose of diagnosis in deep-seated abscess is not to be regarded with favour, as there are dangers of hæmorrhage, peritonitis, etc. In any case, if an exploratory puncture be made, the operation of emptying the abscess should follow immediately. The pus is best evacuated, not by any form of aspiration, but by incision; and since in most cases of hepatic abscess the right lobe is implicated the transthoracic operation is generally required. Two cases are reported by Watts,[4] one of which recovered and the other died after operation.

[1] Brit, Med. Journ., Feb. 8, 1902; Epit., No. 85; and Rec. de Chir., Jan. 10, 1902. [2] Brit. Med. Journ.. Sept. 14, 1901, p. 677. [3] Lancet, Feb 15, 1902, p 460. [4] Brit. Med. Journ., June 29, 1901, p. 1618.

